# Pseudo‐slow–fast atrioventricular nodal reentrant tachycardia: Is the fast pathway a criminal or innocent bystander?

**DOI:** 10.1002/joa3.12955

**Published:** 2023-11-17

**Authors:** Shu Hirata, Koichi Nagashima, Ryuta Watanabe, Yuji Wakamatsu, Yasuo Okumura

**Affiliations:** ^1^ Division of Cardiology, Department of Medicine Nihon University School of Medicine Tokyo Japan

**Keywords:** atrioventricular nodal reentrant tachycardia, bystander, fast pathway, slow pathway

## Abstract

The intracardiac electrograms are shown during scanned single premature ventricular extrastimuli with a decreasing coupling interval in a very short RP tachycardia. What is the diagnosis and is the fast pathway essential for sustaining the tachycardia?

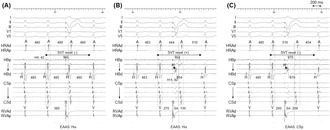

A 69‐year‐old woman with no overt structural heart disease was referred to our hospital to ablate a narrow QRS complex tachycardia. A 12‐lead electrocardiogram during sinus rhythm revealed no preexcitation. Multielectrode catheters were positioned in the high right atrium, His bundle region, coronary sinus (CS), and right ventricular (RV) apex. During sinus rhythm, the atrial–His and His–ventricular intervals were 70 and 47 ms, respectively. Programmed ventricular stimulation with simultaneous atrial and ventricular pacing revealed concentric decremental retrograde conduction with a long HA interval and echo beats, and the earliest atrial activation site (EAAS) was the proximal CS, suggesting multiple atrioventricular nodal pathways without retrograde conduction of fast pathway on baseline testing. The induced clinical tachycardia (SVT1) with a cycle length (TCL) of 560 ms persisted despite sudden VA block occurring. Dissociated sinus rhythm captured the His and QRS but failed to reset the tachycardia (Figure 1). Following an isoproterenol administration, SVT1 transitioned to SVT2, characterized by a 1:1 VA relationship and an EAAS located in the His bundle region. The difference between the post‐pacing interval and tachycardia cycle length corrected by an atrioventricular nodal conduction delay was 158 ms (>110 ms) with a V‐A‐V response after right ventricular overdrive pacing (RVOP), and atrial overdrive pacing (AOP) from a high right atrial site resulted in an A‐H‐A response. The intracardiac electrograms during scanned single premature ventricular extrastimuli with a decreasing coupling interval are shown in Figure 2A–C. What is the diagnosis and is the fast pathway essential for sustaining the tachycardia? Cryothermal energy slow pathway ablation was performed by a fractionated potential‐guided approach with ultrahigh‐resolution mapping. There was no atrioventricular jump or tachycardia post‐ablation.

**FIGURE 1:**
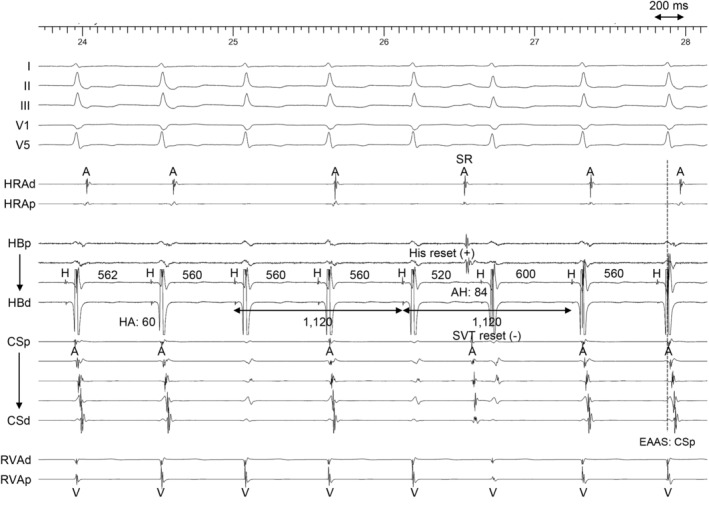
Intracardiac electrogram obtained during supraventricular tachycardia 1. A, atrial; AH, atrial‐His interval; CSd, coronary sinus distal; CSp, coronary sinus proximal; EAAS, earliest atrial activation site; H, His bundle; HA, His‐atrial interval; HBd, His bundle distal; HBp, His bundle proximal; HRAd, high right atrium distal; HRAp, high right atrium proximal; RVAd, right ventricular apex distal; RVAp, right ventricular apex proximal; SR, sinus rhythm; SVT, superior ventricular tachycardia; V, ventricle; VAB, ventricular atrial block.

**FIGURE 2:**
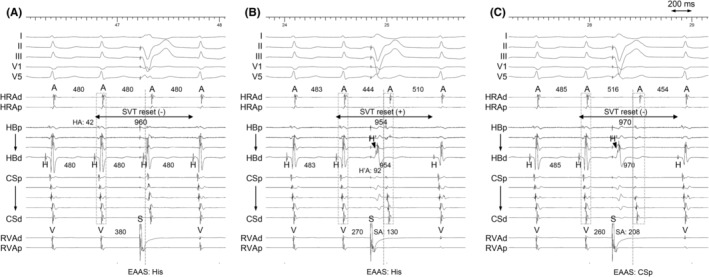
Intracardiac electrograms after scanned ventricular extrastimuli (A) during His bundle refractoriness (B, C) with a decrease in the coupling interval during SVT2. SA, stimulus‐atrial interval. Other abbreviations are as in Figure 1.

The differential diagnosis of the sustained narrow QRS tachycardia despite VA block occurring included intra‐Hisian reentry, upper septal type idiopathic left ventricular tachycardia, junctional tachycardia (JT), orthodromic reciprocating tachycardia (ORT) with a concealed nodoventricular (NV) or nodofascicular (NF) fiber, and atrioventricular nodal reentrant tachycardia (AVNRT) with an upper common pathway.^1^, ^2^ No split His electrograms and identical His–ventricular intervals during sinus rhythm and the SVT1 ruled out intra‐Hisian reentry and idiopathic left ventricular tachycardia. The type of VA block was not Wenckebach VA block, and tachycardia termination during RVOP and AOP was unfavorable for the diagnosis of JT. The lack of SVT1 induction after slow pathway ablation supports this. The dissociated sinus rhythm captured the His and ventricle via the fast pathway but did not affect the tachycardia cycle indicating the His‐Purkinje system and ventricle were not involved in the tachycardia circuit, leading to a diagnosis of AVNRT while excluding ORT involvement with NV or NF fibers. That observation might be a double ventricular response in slow–fast AVNRT representing the HH interval across the dissociated sinus rhythm accidentally identical to twice the tachycardia cycle length, but it is unlikely because no retrograde conduction of the fast pathway was observed on baseline testing. Therefore, also indicated that the anterograde fast pathway conduction was a bystander, leading to the diagnosis of slow–slow AVNRT with an upper common pathway. SVT2 appeared to represent slow–fast AVNRT because the EAAS shifted to the His bundle region with a His–atrial interval <70 ms, and single ventricular extrastimuli during His refractoriness failed to reset the tachycardia (Figure 2A).^3^ Early ventricular extrastimuli reset the SVT without changing the EAAS post‐pacing (Figure 2B), which was consistent with slow–fast AVNRT a diagnosis. However, further decreasing of the ventricular extrastimulus coupling interval exhibited retrograde jump up with a shift in the EAAS toward the proximal CS, indicating retrograde conduction block over the fast pathway. Furthermore, the resetting phenomenon disappeared with this extrastimulus. Those observations indicated that the AVNRT was a slow–slow type, and the retrograde fast pathway was not involved in the tachycardia circuit and was a bystander. The disappearance of tachycardia resetting could possibly have been attributed to the collision of this extrastimulus with the AVNRT in the lower common pathway, which was situated more proximally than the ventricular insertion site of the fast pathway. The 60 ms difference in the His–atrial intervals observed during RV pacing (120 ms) compared to during AVNRT (60 ms) also indicated a lower common pathway was present.^4^ Based on those observations, SVT1 and SVT2 were identical; both the anterograde and retrograde fast pathways mimicked the essential pathway but were bystanders (Figure 3). SVT1 and SVT2 showed different aspects despite the same circuit, because the administration of isoproterenol caused reverse conduction to appear in the fast pathway. We specifically named SVT2, “pseudo‐slow‐fast AVNRT,” because this slow–slow AVNRT mimicked slow–fast AVNRT. Previous reports indicate that the fast pathway does not participate in fast–slow AVNRT's reentrant circuit.^5^ Similarly, there may be slow–fast AVNRT cases where the fast pathway is a bystander and represents a pseudo‐slow–fast AVNRT scenario. It is rare to observe retrograde jump during ventricular extrastimuli, as most slow–fast AVNRTs can be terminated by conduction block over the retrograde fast pathway. Noting retrograde jump in response to early ventricular extrastimuli might be useful for identifying pseudo‐slow–fast AVNRT.

**FIGURE 3:**
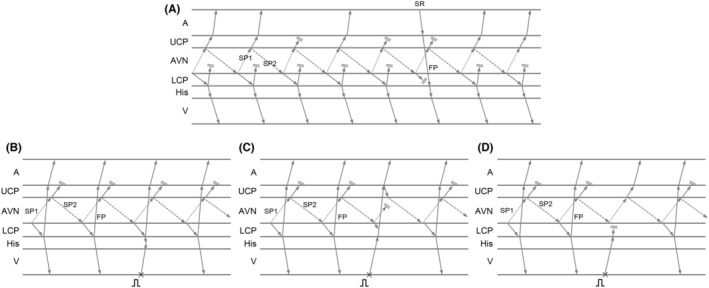
Schematic diagrams corresponding to the electrograms in Figure 1 (A) and Figure 2A–C (B–D). FP, fast pathway; LCP, lower common pathway; UCP, upper common pathway. Other abbreviations are as in Figure 1.

## CONFLICT OF INTEREST STATEMENT

Authors declare no conflict of interests for this article.

## PATIENT CONSENT STATEMENT

The patient has provided consent for publication.
